# Detection of OXA-48-type carbapenemase-producing Enterobacteriaceae in diagnostic laboratories can be enhanced by addition of bicarbonates to cultivation media or reaction buffers

**DOI:** 10.1007/s12223-014-0349-8

**Published:** 2014-09-29

**Authors:** Vendula Studentova, Costas C. Papagiannitsis, Radoslaw Izdebski, Yvonne Pfeifer, Eva Chudackova, Tamara Bergerova, Marek Gniadkowski, Jaroslav Hrabak

**Affiliations:** 1 Department of Microbiology, Faculty of Medicine and University Hospital in Plzen, Charles University in Prague, Alej Svobody 80, 304 60 Plzen, Czech Republic; 2 National Medicines Institute, Warsaw, Poland; 3 Robert Koch Institute, Nosocomial Pathogens and Antibiotic Resistance, Wernigerode, Germany

## Abstract

Carbapenemase-mediated resistance to carbapenems in Enterobacteriaceae has become the main challenge in the treatment and prevention of infections recently. The partially unnoticed spread of OXA-48-type carbapenemase producers is usually assigned to low minimum inhibitory concentrations (MICs) of carbapenems that OXA-48-producing isolates often display. Therefore, there is an urgent need of specific and sensitive methods for isolation and detection of OXA-48 producers in clinical microbiology diagnostics. The influence of bicarbonates on carbapenem MICs against carbapenemase-producing Enterobacteriaceae was tested. We also checked whether the addition of bicarbonates to liquid media supplemented with meropenem may facilitate the selective enrichment of various carbapenemase producers in cultures. Furthermore, the sensitivity of carbapenemase confirmation by matrix-assisted laser desorption/ionization mass spectrometry (MALDI-TOF MS) and spectrophotometric hydrolysis assays upon the addition of NH_4_HCO_3_ was examined. The addition of NaHCO_3_ significantly increased MICs of ertapenem and meropenem for OXA-48 producers. Furthermore, liquid media supplemented with NaHCO_3_ and meropenem were reliable for the selective enrichment of carbapenemase producers. The presence of NH_4_HCO_3_ in buffers used in the spectrophotometric and MALDI-TOF MS carbapenemase detection increased the sensitivity of that assay. Our results demonstrate that bicarbonates in media or reaction buffers can enhance the sensitivity of screening methods and diagnostic tests for carbapenemase producers.

## Introduction

Resistance to antimicrobial agents of pathogenic bacteria has become a major problem of current medicine and epidemiology of infections that could limit the development of new procedures in intensive care, hematooncology, surgery, etc., due to the lack of new active drugs. Over the last decades, carbapenems have been used as the last-resort drugs in the treatment of serious nosocomial infections caused by multidrug-resistant Gram-negative bacteria. Today, resistance to carbapenems has been reported all over the world (Tzouvelekis et al. 2012; Munoz-Price et al. 2013). The most important mechanism is the production of carbapenem-inactivating β-lactamases (carbapenemases) which hydrolyze the amide bond of the β-lactam ring in diverse β-lactam molecules. According to the classification first proposed by Ambler, carbapenemases have been found in the β-lactamase classes A, B, or D (Bush et al. 1995).

The carbapenem-hydrolyzing class D β-lactamases (CHDLs; OXA-types) have been mainly observed in *Acinetobacter* spp. In 2001, the first CHDL- (OXA-48)-producing *Klebsiella pneumoniae* was identified in Turkey, and since then, Enterobacteriaceae with OXA-48 types have spread extensively in Middle East, North Africa, and India (Poirel et al. 2004, 2012; Glasner et al. 2013). In Europe, OXA-48-type-producing Enterobacteriaceae (including variants OXA-48, OXA-162, OXA-163, OXA-181, OXA-204, OXA-232, OXA-244, OXA-245, OXA-247, etc.) have been found in many countries, often due to imports from endemic regions, and their regional spread was reported, for example, in Spain, France, Belgium, the UK, and Ireland (Poirel et al. 2012; Tzouvelekis et al. 2012; Glasner et al. 2013). The minimal inhibitory concentrations (MICs) of carbapenems against OXA-48-type producers range between 0.5 and ≥64 mg/L for ertapenem, 1 and ≥64 mg/L for imipenem, and 1 and ≥64 mg/L for meropenem (Poirel et al. 2012). The OXA-48-type producers with low MICs, categorized as susceptible to carbapenems by the EUCAST and CLSI guidelines, are very difficult to detect and may spread unnoticed, evolving then to resistance phenotypes (Poirel et al. 2012; EUCAST 2013a; CLSI 2013). Therefore, selective isolation and sensitive detection of OXA-48-type producers remain a big challenge for current microbiological diagnostics.

Previous studies have shown that the most important factor of the activity of OXA-type β-lactamases is the carboxylated lysine residue in the active site (Leonard et al. 2008; Docquier et al. 2009; Vercheval et al. 2010). In 2008, Leonard et al. (2008) reported that addition of bicarbonate enhanced the activity of OXA-1 β-lactamase against ampicillin. MICs of ampicillin increased more than 16-fold, whereas no significant changes were observed on the MICs of cephalotin, cefotaxime, ceftazidime, and cefepime that are, however, poor substrates of OXA-1 enzyme. Similar results were described also by Verma et al. (2011), demonstrating that supplementation of the hydrolysis reaction buffer with 50 mM sodium bicarbonate (NaHCO_3_) increased the *k*_cat_/*K*_*m*_ values of the CHDL OXA-58 for ampicillin, amoxicillin, cephalotin, and imipenem by more than five times. The recent studies (June et al. 2014; Che et al. 2014) showed a different behavior of class D enzymes and their ability for dexarboxylation/recarboxylation of active-site lysine.

Based on those results, we tested the effect of NaHCO_3_ as a supplement in the cultivation media on the MICs of carbapenems against OXA-48-type producers in order to check whether this might increase their sensitivity in the detection of these organisms. We also tested if the addition of ammonium bicarbonate (NH_4_HCO_3_) could improve the sensitivity of the MALDI-TOF MS meropenem hydrolysis assay for the detection of OXA-48-producing bacteria.

## Materials and methods

### Bacterial isolates

Previously characterized Enterobacteriaceae isolates from collections of the Department of Microbiology of the Faculty of Medicine and University Hospital in Plzen, Czech Republic, the National Medicines Institute in Warsaw, Poland, and the Robert Koch Institute in Wernigerode, Germany, were used in the study (Pfeifer et al. 2012). The group included 14 OXA-48- and 3 OXA-162-producing clinical isolates, six *Escherichia coli* J53 transformants or transconjugants with OXA-48, 11 clinical isolates producing carbapenemases of the KPC, VIM, or NDM types, nine carbapenemase-non-producing clinical isolates resistant to at least one carbapenem, nine carbapenemase-negative carbapenem-susceptible clinical isolates, and six *E. coli* ATCC 25922 recombinant strains with cloned β-lactamase genes, as mentioned below (Table 1) (Empel et al. 2010; Hrabak et al. 2009, 2011, 2013; Chudackova et al. 2010; Papagiannitsis et al. 2013). *E. coli* ATCC 25922 and *E. coli* ATCC 14169 were used as controls in susceptibility testing and the MALDI-TOF MS meropenem hydrolysis assay, respectively.

**Table 1:** Susceptibility of *E. coli* ATCC25922 transformed with the pTrcHis TOPO® TA vector carrying the cloned β-lactamase genes

β-Lactamase/strain	MIC [mg/L]											
	Ceftazidime	Ceftazidime, BIC	Cefepime	Cefepime, BIC	Ertapenem	Ertapenem, BIC	Imipenem	Imipenem, BIC	Meropenem	Meropenem, BIC	Doripenem	Doripenem, BIC
ATCC25922	0.125	0.125	0.064	0.032	0.016	0.032	0.125	0.064	0.016	0.016	0.016	0.016
TEM-1	0.5	0.25	**0.25**	**0.064**	0.064	0.125	0.125	0.125	0.032	0.032	0.032	0.016
DHA-1	>32	>32	0.25	0.125	**0.064**	**0.25**	0.125	0.064	0.016	0.064	0.032	0.016
OXA-1	0.5	0.25	1	0.5	0.064	0.125	0.125	0.125	0.016	0.032	0.016	0.016
OXA-9	0.25	0.25	**0.25**	**0.064**	0.032	0.125	0.125	0.125	0.032	0.032	0.032	0.016
OXA-48	0.5	0.25	**0.5**	**0.125**	**2**	**16**	**1**	**0.125**	**0.25**	**2**	0.25	1
KPC-2	>32	>32	2	1	0.5	2	0.5	0.25	0.25	0.5	0.125	0.5

Minimal inhibitory concentration determined by microdilution method with (*BIC*) and without sodium bicarbonate (50 mmol/L) and 1 mmol/L IPTG as an inducer. Significant changes are in bold

*MIC* minimal inhibitory concentration

### Preparation of cultivation media

MH broth (Oxoid Ltd., Brno, Czech Republic) supplemented with NaHCO_3_ was prepared as follows: after sterilization (121 °C, 15 min), the media were cooled down to 4 °C, and NaHCO_3_ (50 mmol/L; Sigma-Aldrich Co., Prague, Czech Republic), and meropenem (1 mg/L; AstraZeneca UK Ltd., Macclesfield, UK) were added.

### Cloning of β-lactamases

*bla*_OXA-1_, *bla*_OXA-9_, *bla*_OXA-48_, , *bla*_KPC-2_, *bla*_TEM-1_, and *bla*_DHA-1_ β-lactamase genes were cloned using pTrcHis TOPO® TA Expression Kit (Life Technologies, Prague, Czech Republic) according to the manufacturer’s recommendations. Oligonucleotides for the PCRs followed by cloning were as follows: *bla*_OXA-1_, forward: ATGAAAAACACAATACATATCAACTTCG, reverse: TTATAAATTTAGTGTGTTTAGAATGGTG; *bla*_OXA-9_, forward: ATGAAAAAAATTTTGCTGCTGCATATG, reverse: CACATATCATTTGTTACCCATC; *bla*_OXA-48_, forward: ATGCGTGTATTAGCCTTATC, reverse: CTAGGGAATAATTTTTTCCT; *bla*_DHA-1_, forward: GTTTGTTCTGTCCGG, reverse: TTATTCCAGTGCACTCA; *bla*_TEM-1_, forward: ATGAGTATTCAACATTTCCGT, reverse: TTACCAATGCTTAATCAGTGA; *bla*_KPC-2_, forward: ATGTCACTGTATCGCC, reverse: GATTTTCAGAGCCTTAC. Plasmids were isolated using the Qiagen Plasmid Maxi Kit (Qiagen GmbH, Hilden, Germany) and chemically transformed to *E. coli* ATCC25922 competent cells prepared by the method described by Fournet-Fayard et al. (1995). Transformants were selected on Mueller-Hinton agar containing 50 mg/L of ampicillin.

### Antimicrobial susceptibility testing

MICs of ceftazidime, cefepime, ertapenem, imipenem, meropenem, and doripenem in the presence and absence of NaHCO_3_ (12.5, 25, and 50 mmol/L) were determined by the broth microdilution method, according to the EUCAST recommendations (EUCAST 2003; EUCAST 2013b). Susceptibility of *E. coli* ATCC25922 transformed with plasmids carrying cloned β-lactamase genes was analyzed in MH broth containing 1 mmol/L isopropylthio-β-galactoside (IPTG) as an inducer for the expression of cloned genes (Life Technologies, Prague, Czech Republic). Significant change of MIC was defined as more than twofold dilution step difference.

### Bacterial enrichment in selective MH broth with and without NaHCO_3_

Five milliliters of MH broth supplemented with 1 mg/L of meropenem was inoculated with 10 μl of an inoculum (cell concentration corresponding to 0.5 McFarlands). Inoculum was prepared in 0.9 % saline, using a fresh overnight culture on the Columbia 5 % sheep blood agar (Oxoid CZ, Brno, Czech Republic). After cultivation of inoculated media (35 °C for 18 h), absorbance was measured, using a Jenway 6305 spectrophotometer (Jenway, Staffordshire, UK) at 600 nm. In order to check whether the addition of NaHCO_3_ influences the pH of the medium during cultivation, 18-h cultures were centrifuged (15,000×*g*, 5 min), and the pH of the supernatant was measured using a GMH3530 digital pH meter (Greisinger Electronic GmbH, Regenstauf, Germany).

### MALDI-TOF MS detection of carbapenemase activity

The assay was performed as previously described with slight modifications (Hrabak et al. 2012). A bacterial cell suspension was prepared in the suspension buffer (20 mmol/L Tris–HCl; 20 mmol/L NaCl, pH 7.0) with an addition of 50 mmol/L NH_4_HCO_3_. The reaction was performed in Tris–HCl buffer supplemented with 50 mmol/L NH_4_HCO_3_ (pH adjusted by HCl to 7.0). The incubation time was as previously described (2 h) (Hrabak et al. 2012). For the measurement, 1 μl of the sample was applied on a target (Bruker Daltonics GmbH, Bremen, Germany; MSP 96 Target, Catalog Nr. 224989) and allowed to dry. The spot was then covered by the matrix solution [10 mg/mL of 2,5-dihydroxybenzoic acid and 0.1 μmol/L reserpine in 50 % ethanol] and measured after drying in a range between 350 and 700 mass to charge ratio (*m/z*). All chemicals except meropenem (Astra Zeneca UK) were obtained from Sigma-Aldrich Co., Prague, Czech Republic. Similar measurement was performed using ceftazidime (GlaxoSmithKline, Prague, Czech Republic) as an indicator β-lactam.

### Spectrophotometric detection of carbapenem hydrolysis

For spectrophotometric activity measurement, isogenic *E. coli* ATCC25922 with cloned β-lactamases were used. Fifty milliliters of an overnight bacterial culture in MH broth was centrifuged, resuspended in 500 μl of 1 % glycine (Sigma-Aldrich), and sonicated on ice for 2 × 30 s using a UP50H Ultrasonic Processor (Hielscher–Ultrasound Technology, Teltow, Germany). After centrifugation at 15,000×*g* for 5 min, the supernatant was used for further experiments. The reaction was performed in 50 mmol/L HEPES buffer (pH 7.5) and 50 mmol/L ertapenem, meropenem, imipenem, or doripenem. Five microliters of the crude extract was added to 200 μl of the reaction buffer without or with NaHCO_3_ (25, 50 mmol/L) and measured, using an Infinite® M200 PRO spectrophotometer (Tecan Austria GmbH, Grödig, Austria) at 300 nm (Laraki et al. 1999). Activity was calculated as a slope of absorbance decrease in time.

## Results

### Influence of bicarbonates on antimicrobial susceptibility test results

The effect of NaHCO_3_ on MICs was firstly analyzed using a set of isogenic *E. coli* ATCC25922 recombinant strains, carrying several β-lactamase genes cloned into the expression vector pTrcHis TOPO® TA. As shown in Table 1, significant MIC-increasing effect has been observed in OXA-48-producing isogenic strain for ertapenem (from 2 to 16 mg/L) and meropenem (from 0.25 to 2 mg/L). An MIC increase in low values was also observed for ertapenem (from 0.064 to 0.25 mg/L) in DHA-1-producing isogenic strain. MIC of ertapenem (from 0.5 to 2 mg/L) and doripenem (from 0.125 to 0.5 mg/L) increased in KPC-2-producing strain. A significant MIC decrease has been detected for cefepime in TEM-1- and OXA-48-producing strains. Imipenem MIC significantly decreased in isogenic OXA-48-producer.

**Fig. 1:**
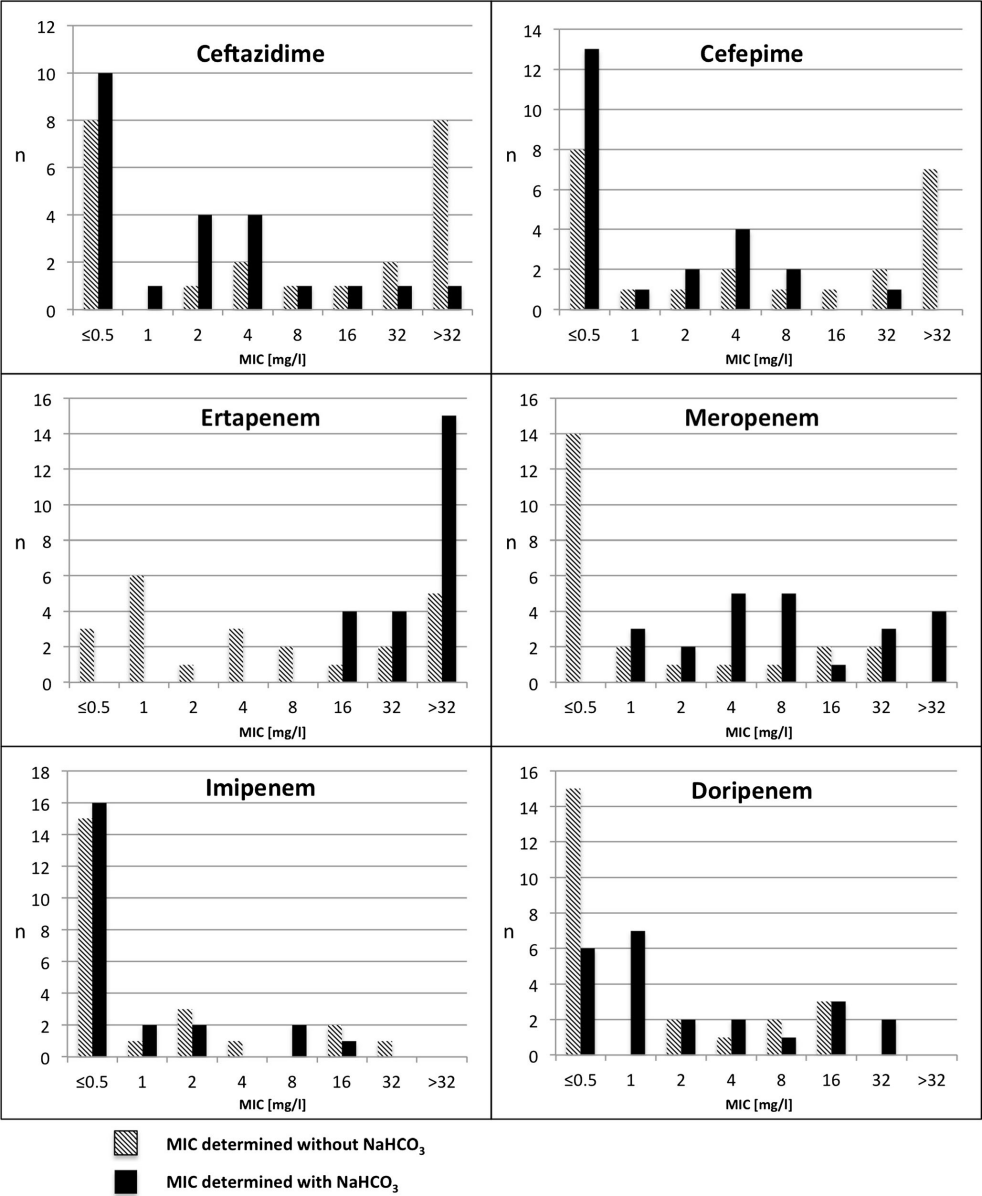
Distributions of MICs of cephalosporins and carbapenems of OXA-48-producing bacteria (*n* = 23) determined by microdilution broth method not supplemented and supplemented with sodium bicarbonate (50 mmol/L)

The comparison of carbapenem and cephalosporin MICs in clinical isolates and transformants/transconjugants revealed the similar behavior comparing with isogenic strains with cloned β-lactamases (Table 2, Fig. 1). For the 23 clinical isolates producing OXA-48-type carbapenemase and their *E. coli* recombinants, the ertapenem MICs increased from ≤0.5–>32 to 16–>32 mg/L in the NaHCO_3_ presence. For meropenem, the effect was more moderate; however, all of the organisms showed remarkably higher MICs upon NaHCO_3_, especially all of the 14 strains with the lowest MIC values of ≤0.5 mg/L. In contrast, the MICs of imipenem remained the same or even decreased for two isolates. For doripenem, the results were ambiguous, with increase, decrease, or no effect in some isolates. NaHCO_3_ addition results in a decrease of MICs of ceftazidime and cefepime.

**Table 2:** Susceptibility of selected isolates tested in Mueller-Hinton broth (strains with same behavior were excluded; the data are presented in Fig. 1)

Isolate	Species	β-Lactamase	MIC ^WO^						MIC, ^BIC^ [mg/L]					
			Ceftazidime		Cefepime		Ertapenem		Meropenem		Imipenem		Doripenem	
ATCC25922	*E. coli*	–	0.125	0.125	0.064	0.032	0.016	0.032	0.016	0.016	0.125	0.064	0.016	0.016
OXA-48-type producers														
OXA_N1	*Citrobacter freundii*	OXA-162, SHV-5	**>32**	**2**	**4**	**≤0.5**	**4**	**>32**	**0.5**	**4**	0.5	≤0.5	**≤0.5**	**2**
OXA_N2	*Enterobacter cloacae*	OXA-48, TEM-1, CTX-M-15	**>32**	**8**	**32**	**4**	>32	>32	32	>32	16	8	16	16
OXA_N3	*E. coli*	OXA-162, TEM-1	0.5	≤0.5	1	≤0.5	>32	>32	**16**	**>32**	4	2	8	16
OXA_N4	*E. coli*	OXA-48, TEM-1, OXA-1	4	1	16	4	>32	>32	32	>32	32	8	**16**	**1**
OXA_N5	*E. coli*	OXA-48, TEM-1, OXA-1	**32**	**4**	**32**	**2**	**4**	**>32**	**0.5**	**8**	0.5	≤0.5	≤0.5	1
OXA_N6	*E. coli*	OXA-48, TEM-1, OXA-1	0.5	≤0.5	0.5	≤0.5	**0.5**	**32**	**≤0.5**	**2**	≤0.5	≤0.5	≤0.5	≤0.5
OXA_C3	*K. pneumoniae*	OXA-48, SHV-1	4	4	**16**	**1**	**1**	**>32**	**≤0.5**	**8**	≤0.5	≤0.5	≤0.5	1
OXA_N9	*Raoultela ornithinolytica*	OXA-162, TEM-1, OXA-1, SHV-5	**>32**	**1**	**2**	**≤0.5**	**4**	**>32**	**0.5**	**4**	0.5	≤0.5	**≤0.5**	**4**
Other carbapenemase-producing isolates														
KPC_C1	*K. pneumoniae*	KPC-2, OXA-9, TEM-1, SHV-12	>32	>32	16	16	>32	>32	**8**	**>32**	8	2	4	2
KPC_C2	*K. pneumoniae*	KPC-3, OXA-9, TEM-1, SHV-12	>32	>32	>32	16	>32	>32	>32	>32	8	1	32	32
KPC_C3	*K. pneumoniae*	KPC-3, OXA-9, TEM-1, SHV-12	8	16	8	1	**1**	**>32**	**≤0.5**	**8**	≤0.5	≤0.5	**≤0.5**	**8**
NDM_B1	*E. coli*	NDM-1, TEM-1	>32	16	>32	4	16	16	4	4	2	0.5	**8**	**32**
NDM_B2	*Citrobacter freundii*	NDM-1, TEM-1, intrinsic AmpC	32	32	16	8	>32	>32	**8**	**>32**	4	2	16	2
NDM_B3	*Providencia stuartii*	NDM-1, intrinsic AmpC	>32	>32	8	8	**4**	**16**	**4**	**16**	8	16	4	2
VIM_C1	*K. pneumoniae*	VIM-1, SHV-1	>32	>32	>32	8	>32	>32	>32	>32	16	1	>32	>32
VIM_C2	*K. pneumoniae*	VIM-1, SHV-1	>32	16	>32	8	**4**	**>32**	**1**	**8**	1	≤0.5	2	2
VIM_C3	*K. pneumoniae*	VIM-1, SHV-1	>32	>32	32	32	**4**	**>32**	**4**	**32**	2	2	**4**	**16**
VIM_C4	*K. pneumoniae*	VIM-4, CMY-4	>32	>32	>32	16	>32	>32	>32	32	8	2	>32	8
Non-carbapenemase-producing carbapenem resistant isolates														
DHA_C1	*K. pneumoniae*	DHA-1, TEM-1, OXA-1	>32	32	>32	16	>32	>32	**2**	**32**	**2**	**8**	2	4
DHA_C2	*K. pneumoniae*	DHA-1, TEM-1, OXA-1, SHV-5	>32	>32	**>32**	**8**	>32	>32	8	16	4	4	4	1
DHA_C3	*K. pneumoniae*	DHA-1, TEM-1, OXA-1	>32	>32	2	2	>32	16	4	4	1	≤0.5	2	0.5
SHV_C1	*K. pneumoniae*	TEM-1, SHV-1, SHV-5	**>32**	**8**	>32	16	>32	32	4	4	1	≤0.5	**2**	**≤0.5**
SHV_C2	*K. pneumoniae*	TEM-1, SHV-1, SHV-5	**>32**	**4**	>32	16	**8**	**>32**	2	4	2	1	1	0.5
Carbapenem susceptible isolates														
ESBL_P1	*E. coli*	TEM-1, OXA-1, CTX-M-15	**32**	**2**	**16**	**1**	**≤0.5**	**16**	≤0.5	≤0.5	≤0.5	≤0.5	≤0.5	≤0.5
ESBL_P2	*K. pneumoniae*	TEM-1, SHV-1, SHV-5	32	16	4	≤0.5	**≤0.5**	**2**	≤0.5	≤0.5	≤0.5	≤0.5	≤0.5	≤0.5
ESBL_P3	*K. pneumoniae*	TEM-1, SHV-1, SHV-5	**>32**	**8**	**2**	**≤0.5**	**≤0.5**	**4**	≤0.5	≤0.5	≤0.5	≤0.5	≤0.5	≤0.5
ESBL_P4	*K. pneumoniae*	TEM-1, SHV-1, SHV-12	>32	16	**2**	**≤0.5**	**≤0.5**	**2**	≤0.5	≤0.5	≤0.5	≤0.5	≤0.5	≤0.5
ESBL_P5	*Providencia stuartii*	TEM-92, intrinsic AmpC	16	ND	8	ND	≤0.5	ND	≤0.5	ND	≤0.5	ND	≤0.5	ND

*MIC* minimal inhibitory concentration, *MIC,* ^*WO*^ minimal inhibitory concentration determined without NaHCO_3_, *MIC,* ^*BIC*^ MIC determined in presence of 50 mmol/L NaHCO_3_, *ND* no growth observed

Significant changes are in bold

The analysis of metallo-β-lactamase (MβL)-producing (VIM, NDM) and KPC-producing bacteria (Table 2) revealed no changes of MICs of doripenem and imipenem. The addition of NaHCO_3_ increased the meropenem MICs for KPC-producing bacteria and for some MβL-producing bacteria, and a significant increase of ertapenem MICs was found for both KPC and MβL producers.

Among the carbapenemase-negative carbapenem-resistant isolates (Table 2), a significant increase in meropenem MIC was observed for one DHA-1 producer and ertapenem MIC for one extended-spectrum β-lactamase (ESβL; SHV-5) producer. For the carbapenem-susceptible ESβL-producing bacteria, no change of MICs of meropenem, imipenem, and doripenem was observed after addition of NaHCO_3_. Only ertapenem MICs increased from ≤0.5 to 1–4 mg/L.

In Table 3, MICs of selected strains in different concentrations of NaHCO_3_ are summarized. No significant MIC increase of ceftazidime and cefepime was observed in all isolates. The highest MIC increase has been detected in ertapenem and meropenem in OXA-48-producing strains using 50 mmol/L NaHCO_3_ that is in concordance with the results described in Table 2 and Fig. 1. For MβL-, KPC-2-, and DHA-1-producing bacteria, carbapenem MICs increase ambiguously. For meropenem, the most significant effect has been observed in 25 mmol/L NaHCO_3_.

**Table 3:** Influence of different NaHCO_3_ concentration on minimal inhibitory concentration (MIC) of selected strains

	Ceftazidime				Cefepime				Ertapenem				Meropenem				Imipenem				Doripenem			
Concentration of NaHCO_3_ (mmol/L)	0	12.5	25	50	0	12.5	25	50	0	12.5	25	50	0	12.5	25	50	0	12.5	25	50	0	12.5	25	50
Strain nr.																								
OXA_N3	0.5	≤0.5	0.5	≤0.5	1	≤0.5	≤0.5	≤0.5	8	32	>32	>32	2	4	16	>32	4	0.5	≤0.5	2	1	1	8	16
OXA_C3	4	1	4	4	16	2	1	1	1	16	32	>32	≤0.5	2	4	8	≤0.5	≤0.5	1	≤0.5	≤0.5	1	2	2
KPC_C1	>32	>32	>32	>32	16	16	32	16	>32	>32	>32	>32	8	32	>32	>32	8	8	8	2	4	8	16	8
VIM_C2	>32	16	16	16	32	16	16	8	4	32	>32	>32	1	8	8	8	1	1	1	≤0.5	2	2	2	2
NDM_B1	>32	>32	>32	16	>32	32	32	4	16	>32	>32	16	4	32	32	4	2	8	2	0.5	8	16	16	2
DHA_C3	>32	>32	>32	>32	2	2	2	2	8	>32	>32	>32	2	8	16	4	2	8	16	1	1	2	4	0.5
SHV_C2	>32	32	16	4	>32	>32	>32	16	>32	>32	>32	32	4	4	4	4	1	1	1	≤0.5	2	2	2	≤0.5

### Selective enrichment in broth supplemented by sodium bicarbonate

Based on the data mentioned above, the most specific increase of MICs for OXA-48 producing bacteria has been observed for meropenem. Therefore, this antibiotic was used for further experiments. In this step, we tried to optimize a method for increasing the sensitivity of selective media for the detection of OXA-48-type-producing bacteria. The results are summarized in Table 4. Using the MH broth supplemented with 1 mg/L meropenem and 50 mmol/L NaHCO_3_, we found 100 % sensitivity for the OXA-48-type-producing bacteria used in the study, including all those with low MICs of meropenem (≤0.5 mg/L). No growth was observed in the case of ESβL-producing and AmpC-producing isolates with meropenem MICs of ≤0.5 mg/L. Bacterial growth was detected for all of the MβL and KPC producers used, and all of the carbapenemase-non-producing bacteria with elevated meropenem MICs (≥1 mg/L).

**Table 4:** Test of liquid screening cultivation media supplemented with meropenem (1 mg/L) and NaHCO_3_ (50 mmol/L) after 18 h cultivation at 35 °C

Isolate	A_600_	
	without NaHCO_3_	with NaHCO_3_
ATCC25922	0.079	0.054
OXA-48-type producers		
OXA_N1	0.353	0.851
OXA_N2	0.129	1.000
OXA_N3	0.950	1.100
OXA_N4	0.926	1.050
OXA_N5	0.104	1.050
OXA_N6	0.079	0.751
OXA_C3	0.029	1.000
OXA_N9	0.751	1.100
Other carbapenemase-producing isolates		
KPC_C1	0.701	1.150
KPC_C2	0.901	1.150
KPC_C3	0.652	1.025
NDM_B1	0.876	0.926
NDM_B2	0.751	0.776
NDM_B3	0.502	0.851
VIM_C1	0.776	1.224
VIM_C2	0.253	0.776
VIM_C3	0.627	0.826
VIM_C4	0.502	1.000
Non-carbapenemase-producing carbapenem-resistant isolates		
DHA_C1	0.826	1.100
DHA_C2	0.851	1.174
DHA_C3	0.328	0.403
SHV_C1	0.726	0.851
SHV_C2	0.552	0.950
Carbapenem susceptible isolates		
ESBL_P1	0.029	0.079
ESBL_P2	0.079	0.079
ESBL_P3	0.104	0.154
ESBL_P4	0.129	0.129
ESBL_P5	0.079	0.104

Data of selected strains are presented (strains with similar behavior have been excluded)

In water solution, sodium bicarbonate is decomposed to sodium carbonate, water, and carbon dioxide. Therefore, we checked its effect on pH of the liquid cultivation media. In the cultures with bacterial growth observed, pH of the media supplemented with NaHCO_3_ remained in the interval of 6.74 to 7.1 (median 6.79), contrary to the cultures grown in the MH broth without NaHCO_3_, where acidification of the medium was observed (pH, 5.73–7.24; median, 5.98). The media supplemented with NaHCO_3_, where no bacterial growth was detected, had pH values ranging from 7.6 to 7.7. These findings allowed us to conclude that neither carbapenem molecules nor bacterial cells are affected during cultivation in bicarbonate-supplemented media due to alkalization.

### MALDI-TOF MS meropenem hydrolysis assay

**Fig. 2:**
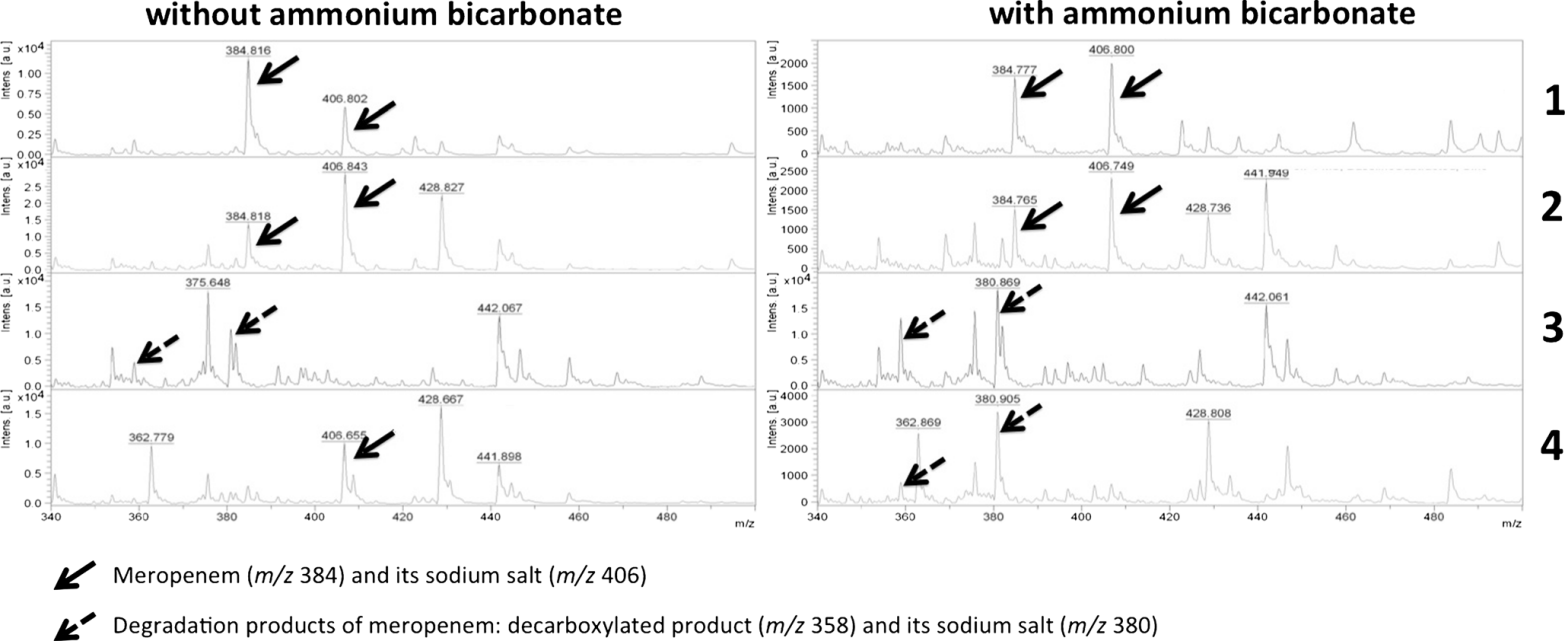
Results of MALDI-TOF MS meropenem hydrolysis assay in absence and presence of 50 mmol/L ammonium bicarbonate. *1* meropenem, *2* ATCC 14169 (negative control), *3* KPC_C1–KPC-2-producing *K. pneumoniae*; *4* OXA_C3–OXA-48-producing *K. pneumoniae*

For the OXA-48-type-producing clinical isolates or recombinants with low carbapenem MICs (*n* = 13), the MALDI-TOF MS hydrolysis assay was performed as described previously (Hrabak et al. 2012), yielding no positive results (Fig. 2). After addition of NaHCO_3_ to the reaction buffer, we were not able to obtain readable spectra; therefore, we used 50 mmol/L NH_4_HCO_3_. In the reaction with NH_4_HCO_3_, meropenem and its sodium salt (*m/z* 384 and *m/z* 406) were not detected, while meropenem degradation products (*m/z* 358 and *m/z* 380) were observed for all of the OXA-48-type-producing strains. In contrast, no complete hydrolysis was detected in the reaction without NH_4_HCO_3_. Sensitivity of the test was not affected by NH_4_HCO_3_ for VIM-, NDM-, or KPC-producing bacteria. In our MALDI-TOF MS data, we did not observe peaks with molecular weight values corresponding to meropenem-CO_2_ or its salts after hydrolysis by carbapenemases (Almarsson et al. 1998).

Using ceftazidime as an indicator antibiotic, a peak representing a ceftazidime molecule with cleaved pyridine (*m/z* 468.6) (Sparbier et al. 2012) remained presented in all strains producing no carbapenemases as well as in OXA-48 strains producing no ESβL. In all strains producing carbapenemases able to hydrolyse ceftazidime, the peak corresponding to non-hydrolyzed ceftazidime disappeared. A peak representing hydrolyzed and decarboxylated molecule (*m/z* 442.6) has been observed in all ESβL-producing strains, as well as all carbapenemase-producing strains, except those solely expressed OXA-48-type enzymes. No influence of NH_4_HCO_3_ has been observed (data not shown).

### Spectrophotometric hydrolysis assay

In order to prove that the increased resistance to meropenem and ertapenem upon the addition of bicarbonates is due to the enhancement of the OXA-48-type carbapenemase activity, we performed the spectrophotometric hydrolysis assay. Data are shown in Table 5. In concordance with MALDI-TOF MS hydrolysis results, an increasing of carbapenemase activity in presence of NaHCO_3_ has been detected in OXA-48 isolates. An increase, however, has been observed in other carbapenemases as well. Interestingly, a slight carbapenemase activity was observed in OXA-1-producing strain against meropenem and imipenem in presence of sodium bicarbonate.

**Table 5:** Activity of crude extracts of isogenic *E. coli* with cloned β-lactamases

	Ertapenem				Meropenem				Imipenem				Doripenem			
Concentration of NaHCO_3_ (mmol/L)	0	25	50	Activity/50 mmol/L	0	25	50	Activity/50 mmol/L	0	25	50	Activity/50 mmol/L	0	25	50	Activity/50 mmol/L
OXA-1	ND	ND	ND	ND	8 %	20 %	100 %	0.00203	25 %	41 %	100 %	0.00358	ND	ND	ND	ND
OXA-9	ND	ND	ND	ND	ND	ND	ND	ND	ND	ND	ND	ND	ND	ND	ND	ND
OXA-48	23 %	36 %	100 %	0.00465	42 %	51 %	100 %	0.00782	32 %	45 %	100 %	0.00680	31 %	51 %	100 %	0.00554
VIM-1	62 %	74 %	100 %	0.00271	44 %	49 %	100 %	0.00848	20 %	43 %	100 %	0.00845	31 %	40 %	100 %	0.00608
KPC-2	63 %	83 %	100 %	0.00292	42 %	60 %	100 %	0.00872	35 %	56 %	100 %	0.00872	31 %	65 %	100 %	0.00437

Activity was determined as a slope of absorbance decrease in time

*ND* no activity against the antibiotic tested was detected (activity index <0.001)

## Discussion

Previous studies showed that bicarbonates, being a source of CO_2_, significantly affect catalytic activity of OXA-type β-lactamases and may raise β-lactam resistance levels in bacteria producing these enzymes (Leonard et al. 2008; Docquier et al. 2009; Vercheval et al. 2010; Verma et al. 2011). Based on the structure, for the activity of OXA-48 β-lactamase, carbo-xylation of active-site Lys is important for the activity of the enzyme (Docquier et al. 2009). In some OXA-type enzymes, different rate of decarboxylation of active-site Lys has been observed for different substrates (Che et al. 2014). That observation could explain different behavior of OXA-48-producing strains against different carbapenems. However, further structural studies are needed. Recently, Antunes et al. (2014) demonstrated that OXA-2 and OXA-10 β-lactamases expressed in *Acinetobacter baumannii* possess extended-spectrum activity also conferring resistance to carbapenems. Expressed in *E. coli*, however, both enzymes possessed a narrow-spectrum activity only. Those results may support our observance of different behavior in presence of bicarbonates in OXA-type enzymes and their activity against carbapenems (see Table 5).

In the present study, we demonstrated that CO_2_ also affect the resistance in ertapenem and meropenem of Enterobacteriaceae with OXA-48-like β-lactamases, which are one of the key types of carbapenemases spreading nowadays worldwide. As we showed using the set of *E. coli* ATCC25922 recombinant clones expressing various β-lactamases, the MICs of ertapenem and meropenem significantly increased against OXA-48 producer in the presence of NaHCO_3_. Some MIC increase was observed also for producers of other β-lactamase types (e.g., KPC-2, DHA-1). This observation could be explained by different behavior of OXA-48 β-lactamase and deacylation of active-site Lys or by some non-specific changes in the cell wall. At this moment, it is impossible to reveal specificity and mechanism of this effect.

For direct detection of carbapenemase activity, two different assays have been recently developed (Nordmann & Poirel 2013). MALDI-TOF MS detection of carbapenem hydrolysis is based on a detection of peaks corresponding with indicator carbapenem molecule and its degradation products in mass spectra. Interestingly, a calibration molecule, reserpine, added in the matrix solution, improved a quality of the spectra suppressing the noise/signal ratio.

By way of contrast, the principle of Carba NP test is a detection of pH decrease during carbapenem hydrolysis using phenol red as an indicator. Our results indicate that addition of bicarbonates to the reaction buffers may significantly increase the sensitivity of MALDI-TOF MS hydrolysis assay. Based on the principle, addition of that component to reaction buffers in Carba NP test may be problematic due to an alkalization of reaction mixture as a result of bicarbonate spontaneous decomposition.

We also demonstrated that sensitivity of screening cultivation media for OXA-48-type carbapenemase-producing bacteria can be enhanced by the addition of bicarbonates. Similar results as for broth have also been observed for solid cultivation media (data not shown). Such media could be used for isolation of carbapenemase-producers from screening samples. However, validation on real clinical specimens is needed.

As demonstrated in some OXA-48-producing strains, no significant growth was observed in presence of 1 mg/L of meropenem. Addition of bicarbonates to the cultivation media clearly enhanced the growth of all OXA-48-type producing bacteria (*n* = 23). Bicarbonates, however, did not improve the specificity of the tests for the detection of OXA-48 carbapenemases, but they significantly improved their sensitivity. This may be due to a better buffering of cultivation media or reaction mixture resulting in efficient re-carboxylation of active-site Lys.

Carbon dioxide is an important component of body fluids as well, either dissolved or in the form of bicarbonate and carbamino-compounds (Barrett et al. 2009). The pathogenic bacteria invading the human organism are surrounded by an environment rich in bicarbonates and carbon dioxide. Consequently, their metabolic activity, including expression of resistance mechanisms, may be influenced by these substances. The standard MH agar and broth used for susceptibility testing contain very low levels of HCO_3_^−^ ions in comparison with its concentration in the organism (normal concentration of HCO_3_^−^ in arterial plasma is between 22 and 29 mol/L) (Burtis 2006). Based on the MIC changes of some carbapenems (ertapenem, meropenem) and cephalosporins in the presence of bicarbonates, we believe that further studies on the development of a more reliable procedure for the determination of bacterial behavior in vivo (i.e., on animal models) should be performed. However, many other factors may be responsible for such MIC changes.

Currently, interpretation criteria for ESβL- and carbapenemase-producing bacteria have been under discussion (Livermore et al. 2012; Nordmann and Poirel 2013). Formerly, ESβL-producing bacteria were interpreted as resistant to all penicillins and cephalosporins, regardless of the MIC values (or inhibition zone diameters). A similar approach was applied for carbapenemase-producing bacteria that often were interpreted as resistant to all carbapenems by definition. Recently, breakpoints for cephalosporins and carbapenems were decreased by both EUCAST and CLSI, and according to their proposals, susceptibility should be reported “as found” based on the MIC or the inhibition zone diameter. ESβLs and carbapenemases should be reported for infection control and epidemiological purposes only; however, this approach has been questioned by some authors, who keep interpreting all carbapenemase producers as resistant to carbapenems, regardless of the in vitro testing data. The latter criteria are discussed in recently published in vivo studies (Wiskirchen et al. 2014a, b). For the infections induced by OXA-48-producing strains, the authors found that the outcome of treatment by ceftazidime and ertapenem of isogenic strains correlated with MIC data (Wiskirchen et al. 2014a). However, poor efficacy was observed for clinical strains. Based on those data, identification of resistance mechanism and carbapenemase type, respectively, may be important for proper antibiotic therapy caused by such bacteria. Contrary to those results, carbapenems seem to be an acceptable choice for NDM-1-producing bacteria while no better drugs or β-lactamase inhibitors are available (Wiskirchen et al. 2014b).

Similarly to mathematical modelling, in vitro susceptibility testing using MIC or IZ diameter determination is only a simplification of the real situation in the organism during an infection. In many clinical studies describing an effect of β-lactams in ESβL- or carbapenemase-producing bacteria, only MIC data are available. Based on the information mentioned above, we also propose that, in those studies, the detailed data on the β-lactamase content should always be provided.
